# Acoustic micro-tapping for non-contact 4D imaging of tissue elasticity

**DOI:** 10.1038/srep38967

**Published:** 2016-12-23

**Authors:** Łukasz Ambroziński, Shaozhen Song, Soon Joon Yoon, Ivan Pelivanov, David Li, Liang Gao, Tueng T. Shen, Ruikang K. Wang, Matthew O’Donnell

**Affiliations:** 1 Department of Bioengineering, University of Washington, Seattle, WA 98195, USA; 2 AGH University of Science and Technology, Krakow, Poland; 3 Faculty of Physics, Moscow State University, Moscow, 119991, Russia; 4 Department of Chemical Engineering, University of Washington, Seattle, WA 98195, USA; 5 Department of Ophthalmology, University of Washington, Seattle, WA 98104, USA

## Abstract

Elastography plays a key role in characterizing soft media such as biological tissue. Although this technology has found widespread use in both clinical diagnostics and basic science research, nearly all methods require direct physical contact with the object of interest and can even be invasive. For a number of applications, such as diagnostic measurements on the anterior segment of the eye, physical contact is not desired and may even be prohibited. Here we present a fundamentally new approach to dynamic elastography using non-contact mechanical stimulation of soft media with precise spatial and temporal shaping. We call it acoustic micro-tapping (AμT) because it employs focused, air-coupled ultrasound to induce significant mechanical displacement at the boundary of a soft material using reflection-based radiation force. Combining it with high-speed, four-dimensional (three space dimensions plus time) phase-sensitive optical coherence tomography creates a non-contact tool for high-resolution and quantitative dynamic elastography of soft tissue at near real-time imaging rates. The overall approach is demonstrated in *ex-vivo* porcine cornea.

For nearly thirty years, elastography has been used to spatially map the shear/Young’s modulus (or elasticity) of soft materials such as biological tissue^1^,^2^,^3^,^4^. Multiple deformation sources (e.g., vibrator, static compressor, air-puff and acoustic radiation force) can create material displacements and numerous imaging systems (e.g., ultrasound (US), magnetic resonance imaging (MRI), and optical coherence tomography (OCT)) can track these displacements leading to spatial maps of the elasticity^1^,^2^,^3^,^4^,^5^,^6^,^7^. Recent work combining phase-sensitive OCT (PhS-OCT) with several different deformation sources created a new technique called optical coherence elastography (OCE) in which propagating shear (or guided) waves are tracked to map the elasticity in the skin and anterior segments of the eye with unprecedented sensitivity and spatial resolution^8^,^9^,^10^.

Unfortunately, nearly all elastography approaches require direct contact with the material under study. For many applications in biomedicine and other fields, however, a totally non-contact system (for both excitation and detection of mechanical waves) is desirable and, in some cases, required. In particular, for OCE to be used routinely in ophthalmology and dermatology, and potentially for biopsy characterization, a robust non-contact technology is needed to produce mechanical waves.

Non-contact mechanical wave generation in soft media has been demonstrated in a limited number of studies. For example, an air-puff produced very narrow bandwidth mechanical waves in the cornea^10^,^11^,^12^,^13^. Due to the long excitation and recovery times of the system, the repetition rate of the source is limited to about 100 Hz, while the typical repetition rate is 50 Hz^12^,^13^. It is also very difficult to spatially shape the air-puff source, a very important feature for quantitative measurements. Given these limitations, it may not be possible to produce high resolution maps of tissue elasticity with an air-puff source.

An alternate non-contact approach has recently been demonstrated in which pulsed ultraviolet (UV) laser light is absorbed at the surface (within tens of μm depth) of the cornea^14^. The mechanical wave bandwidth can easily reach 10 kHz, providing superior lateral resolution in elasticity imaging^14^,^15^. The source can also be easily shaped with a patterned laser excitation. Ultraviolet light is highly absorptive in soft tissue and is, therefore, a very efficient source of compressional acoustic waves. However, laser generation of shear displacement is efficient only when the UV fluence approaches safety limits. Clearly, there is still great need for a non-contact method to generate shear mechanical waves in soft media with the spatial and temporal compactness required for high spatial resolution elasticity imaging.

Recently, we observed that an air-coupled US beam reflected from an air/soft-medium interface can generate significant shear displacement through reflection-based acoustic radiation force (ARF)^16^. Unlike relatively inefficient ARF techniques using acoustic loss and scattering mechanisms^3^,^4^,^17^,^18^,^19^,^20^,^21^,^22^, a reflection-based approach can be a very efficient transducer converting acoustic intensity into shear displacement^16^. Building on this observation, here we describe a fully non-contact, non-invasive and clinically translatable OCE system to quantitatively map soft tissue elasticity at high spatial resolution, and demonstrate its performance using *ex-vivo* measurements on porcine cornea.

Non-contact mechanical excitation is performed with a specially designed piezoelectric transducer launching **through air** an US beam focused onto the air-medium interface. Beam reflection at this interface produces significant ARF toward the medium, inducing a transient displacement at that surface (including a shear one) and ultimately generating a propagating mechanical wave in the lateral (transverse to the surface normal) direction. This action is like a hammer tapping wood or a stick beating a drum where a localized, transient force on the target creates significant displacement in a transverse direction to that force. Indeed, due to the large difference in acoustic impedances of air and soft media such as tissue, the efficiency of acoustic energy conversion approaches one hundred percent. For our case, the transient displacement need only be about one μm and the acoustic pressure only a few kPa, a level far below any potential damage thresholds for tissue and, thus, absolutely non-invasive (**Methods Section**). Given this background, we call the method acoustic micro-tapping (AμT).

For applications in ophthalmology and dermatology, sub-mm resolution elasticity maps will be required. At this resolution, the transient displacement scale, or the wavelength of the excited mechanical wave, should be less than one mm. Consequently, the spatial extent and bandwidth of the mechanical excitation must be sub-mm and multi-kHz, respectively. Here we designed and built a focused air-coupled piezoelectric transducer that can efficiently transfer a 1 MHz US pulse through air to the tissue surface with sufficient acoustic energy to launch a few kHz bandwidth mechanical waves with μm-scale displacements easily captured by a high frame rate OCT system. To our knowledge, this is the first demonstration of efficient excitation of high bandwidth mechanical waves in soft tissue with air-coupled AμT. Details of the transducer design, including spatial, temporal, and amplitude characteristics, can be found in the **Methods Section**.

For true clinical translation of non-contact AμT-OCE, an appropriate PhS-OCT system must track high bandwidth mechanical waves propagating in four dimensions (i.e., three space dimensions plus time – 4D). Recently, we developed a 16 kHz frame rate PhS-OCT system appropriate for three-dimensional (3D) imaging (i.e., 4D data acquisition) over large dimensions. Here we use this system to capture mechanical wave propagation over a tissue volume in a fraction of a second (over 3 Hz volume rate) to reconstruct a 3D elasticity map from a single AμT excitation per plane within the volume. A more detailed description of the PhS-OCT system can be found in the **Methods Section**.

Combining AμT for non-contact transient wave excitation with 4-D PhS-OCT imaging of the propagating mechanical wave yields a single-sided, non-contact method to non-invasively measure the elasticity of soft materials such as biological tissue. The ultimate goal is non-contact, quantitative, 3-D mapping of the Young’s modulus at near real-time rates. System performance is demonstrated below on porcine cornea (**Results Section**) with 4D displacement maps and 3D wave speed reconstructions based on these maps. To our knowledge, these are the first images of their kind.

## Results

### OCE with an AμT source of transient displacement

**Figure 1:**
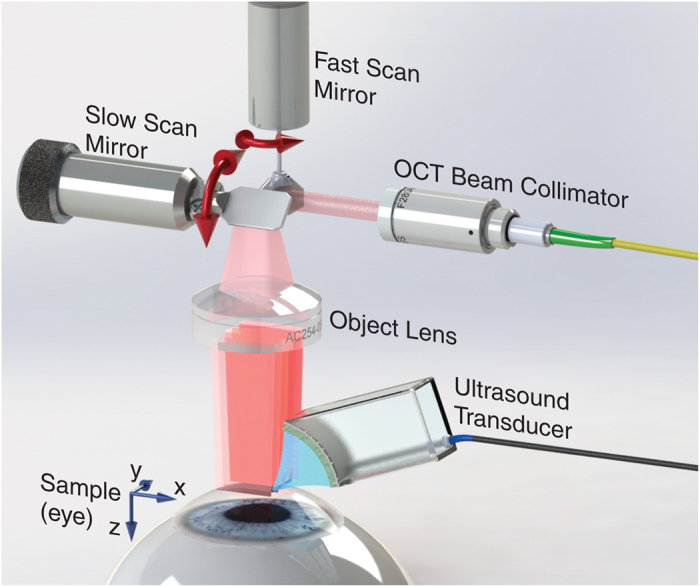
Layout of acoustic micro-tapping and 4D PhS-OCT imaging system for soft tissue elastography. A focused acoustic beam is launched in air with a home-made air-coupled piezoelectric transducer to form a strip acoustic intensity distribution at its focus near the air/medium interface (along Y-axis). The transducer is a cylindrical segment of a piezoelectric tube, 26 mm inner diameter and 30 mm outer diameter (cat. #42–1051, American Piezo International Ltd., Mackeyville, PA 17750 USA), with electrodes on both sides cut to an angle of 75 degrees and length of 9 mm. A matching layer (0.45 μm pore size nylon membrane filter, Cat. No. 7404-004, “GE Healthcare UK Limited”, Little Chalfont, UK) is glued with silicone to its front surface for efficient transfer of US into air at a carrier frequency of 1 MHz. The acoustic intensity field emitted by the transducer can be found in Supplementary Fig. S2. The transducer is tilted to the tissue normal by about 45 degrees to not block the OCT beam. Reflection of the acoustic beam from the air/medium interface produces significant ARF toward the medium, inducing a transient displacement at that surface (including a shear one) and ultimately generating a propagating mechanical wave in the lateral (transverse to the medium normal) direction. We call this effect acoustic micro-tapping (AμT). A 15,900 frame rate PhS-OCT system is used to track mechanical wave propagation with time. Repeated B-Scans are acquired on each imaging (XZ) plane for one AμT pulse, resulting in only 3 ms time to fully track mechanical wave propagation in a (XZ) plane in space and time. Rapid sweeping of the B-Scan plane to another Y position (for 102 different Y positions in total spaced 58.8 μm each other) is used to acquire the entire 4D data set of 102 imaging planes over a 6 × 6 mm lateral field of view in 0.3 s. One AμT firing is performed for a single B-scan. The procedure is repeated 10 times to improve the signal-to-noise ratio, resulting in 3 s total data acquisition time.

Figure 1 describes a method for fully non-contact soft tissue elastography using AμT to excite broadband mechanical waves and 4D tracking of the transverse propagating displacement with an ultra-fast PhS-OCT imaging system. It utilizes an US beam launched with a focused, air-coupled transducer (i.e., propagating through air) to the air/medium interface (**Methods Section**, Supplementary Fig. S1). The US wave reflected from this interface provides acoustic radiation force (ARF) to the medium surface, determined by the spatial shape of the pump beam and the duration of the ultrasound pulse^16^. For pulsed insonification, it “taps” the surface, inducing a transient displacement at that surface and, ultimately, generating a propagating shear/guided/interface/Lamb wave (the mode is determined by boundary conditions). We call this the mechanical wave to maintain generality. A 15,900 frame rate PhS-OCT system (**Methods Section**, Supplementary Fig. S2) tracks mechanical wave propagation. Note that only 3 ms is required to fully track propagation in a (XZ) plane, and only 0.3 sec is needed to acquire time-dependent volumetric data (entire 4D data set) over a 6 mm × 6 mm lateral field of view.

### 4D imaging of mechanical wave propagation in *ex-vivo* porcine eye cornea

The efficient excitation of mechanical waves with an AμT source and their ultra-fast imaging with PhS-OCT produces snapshots of transient displacement at any instant of wave propagation. It takes ~1 ms for a mechanical wave to propagate 6 mm (linear image size in propagating X direction); the PhS-OCT system, however, acquires 16 snapshots per ms. Thus, the propagating wave can be easily captured. Experiments were performed on the cornea from a freshly excised porcine eye at four (10, 20, 30 and 40 mmHg) intraocular pressures (IOP) and four (0°, 45°, 90° and 135°, calculated from X-axis) propagation directions for each IOP resulting in 16 complete 4D image volumes.

**Figure 2:**
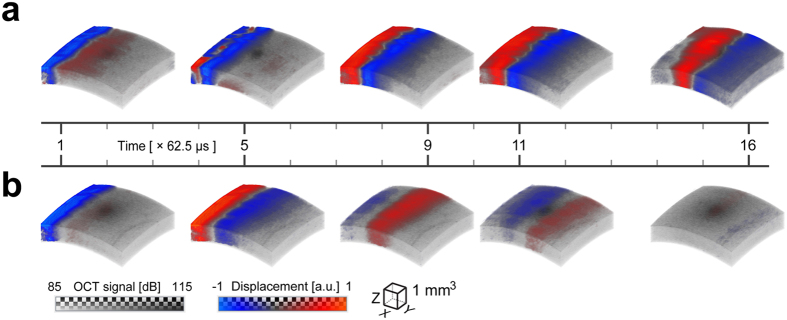
Transient displacement of a mechanical wave propagating in *ex-vivo* porcine eye. A blue-red colormap is used to map the displacement interleaved with the gray-scale of the OCT amplitude image. The bottom of the colorbar indicates the voxel color and the top indicates the transparency applied to any voxel, on a checkerboard background. Five time instants of 3D transient displacement associated with mechanical wave propagation within an *ex-vivo* porcine eye are shown at two different intraocular pressures (**a**) – 10 mmHg, and (**b**) – 40 mmHg for propagation at 0°. The displacement amplitudes are normalized for both data sets by their maxima in the excitation area (62 nm and 34 nm, respectively). The sampling interval between volumes is 62.5 μs, and the time indexes of selected time instants are marked on the timeline. All time instants of wave propagation are shown in Supplementary Movie S3. As seen, the wave propagates much faster for 40 mmHg IOP. At this pressure, the wave already exits the region by the 16^th^ time instant, but for the 10 mmHg case the wave is near the middle of the image area at the same instant.

Figure 2 shows two of these 4D data sets - a group of transient displacement snapshots at 10 mmHg (a) and 40 mmHg (b) pressures for 0° propagation, respectively. The entire sequence is presented in Supplementary Movie S3. As expected, AμT with a cylindrically focused transducer provides a “thin strip” source at the cornea surface. The strip length corresponds very well to the transducer focal zone in the (XY) plane (panel (b) of Supplementary Fig. S1). The strip width determines the characteristic wavelength of the propagating wave and localizes displacements to about 0.5 mm, which also corresponds well to the transducer focal zone in the (XZ) plane (panels (b) and (c) of Supplementary Fig. S1), taking into account an approximately 45 degree transducer tilt with respect to the cornea normal.

Both the length and width of the source determine the character of wave propagation over the cornea. For our case, wavefront curvature does not change over the entire propagation distance and, therefore, can be interpreted as simple plane-wave propagation. Plane waves do not diffract, so diffraction effects can be ignored for wave speed estimation. This is much simpler than the case of spherical waves propagating from a point-like source where frequency-dependent diffraction can be significant. Indeed, the propagating wave in the cornea is leaky, emitting part of its energy deep into the eye. This leakage induces strong frequency dispersion (**Discussion Section**). When frequency dependent diffraction is also present, extracting quantitative information from experimental data can be a challenge. This is especially true if the elastic modulus is estimated from wave velocities.

As is also evident in Fig. 2, the mechanical wave propagates much faster at 40 mmHg IOP. For instance, the wave already exits the region by the 16^th^ time instant, but at 10 mmHg the wave is near the middle of the image at the same instant, indicating increased cornea elasticity with increased IOP.

### Velocity and displacement of mechanical wave

Three-dimensional images of propagating displacements can be used to estimate the wave speed at every point within the volume, and wave speed maps can be used to estimate the elastic modulus non-invasively if the relationship between speed and modulus is well defined for the experimental conditions. The group velocity characterizes the rate of maximum amplitude propagation regardless of the wave harmonic content.

**Figure 3:**
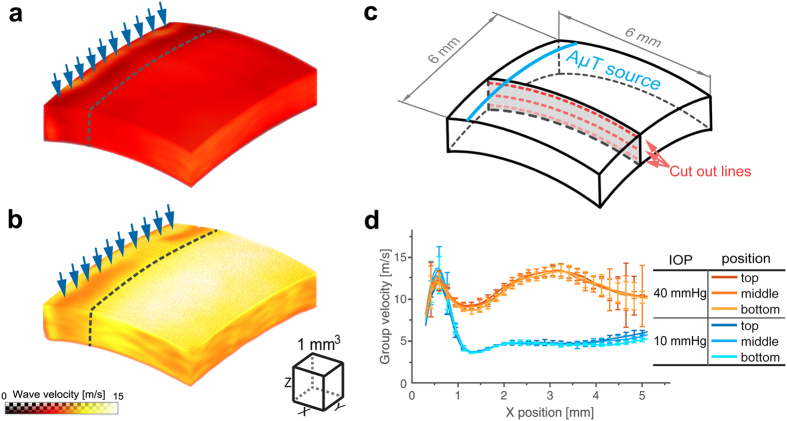
Mechanical wave group velocity in *ex-vivo* porcine eye cornea. 3D maps at two different intraocular pressures (**a**) −10 mmHg, and (**b**) −40 mmHg for propagation at 0°. The group velocity is calculated at each point within the 6 mm × 6 mm lateral field of view using temporal profiles of recorded signals and the phase zero-crossing method applied to the cross-correlation function between neighboring signals with no averaging applied to recorded signals. To create the velocity maps, a moving average procedure is applied to the computed velocity distributions within an effective volume of 294 μm × 294 μm × 114 μm in X, Y and Z directions, respectively. The excitation line is indicated by blue arrows. The dashed line approximately indicates the near field region of wave propagation. Three lines are chosen for different depths of wave propagation in the cornea (close to the top surface, in the middle and close to the bottom of the cornea) as depicted in (**c**). Group velocity on these lines is plotted in (**d**) for both intraocular pressures. High fluctuations of group velocity at distances far from the AμT source are related with reduced signal-to-noise ratio in that region.

Figure 3a,b present 3D distributions of group velocity in an *ex-vivo* porcine eye cornea. They were computed using a cross-correlation-based phase-zero crossing method^23^. Detected signals separated by 6 (six) spatial points along the trajectory X_tr_ (i.e. ΔX_tr_ = 352.8 μm) were cross-correlated to determine the time-lag, Δt_g_, (and, therefore, the group velocity as V_g_ = ΔX_tr_/Δt_g_). The procedure was repeated for all detection points within the volume. Finally, a moving average procedure was applied to the velocity distributions within an effective volume of 294 μm × 294 μm × 114 μm in X, Y and Z directions, respectively.

Figure 3d shows the group velocity versus coordinate X_tr_ for different depths. The group velocity for 10 mmHg IOP at 0° propagation is relatively homogeneous over both propagation distance and depth, except in a near field region (the dashed line in Fig. 3a,b) in which the group speed calculation is incorrect due primarily to artifacts in the OCT signal induced by the ultrasound source. At 40 mmHg IOP and 0° propagation, the group velocity does not change much with depth, but varies with propagation distance. Although a low signal-to-noise ratio for distances larger than 4 mm from the source leads to larger inaccuracies, we cannot guarantee that the group velocity change with distance is insignificant. Possibly, the system maintaining the IOP creates additional cornea thickness and curvature heterogeneities or non-linear elasticity changes for such an artificially high IOP. Such hypotheses will be tested in future studies. Overall, the average group velocity at 40 mmHg is more than twice that at 10 mmHg IOP.

**Figure 4:**
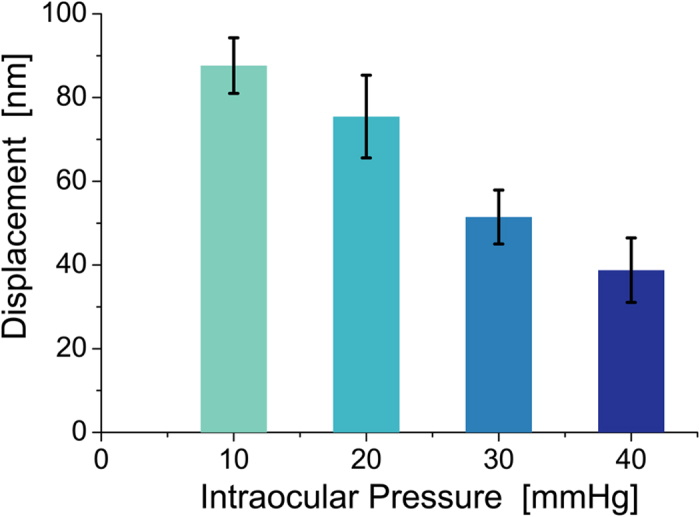
Amplitude of vertical displacement in the propagating mechanical wave versus intraocular pressure (IOP). AμT source and area of OCT scanning were kept the same at all IOPs for propagation at 0°. Error bars indicate RMS fluctuations of displacement amplitude over the excitation area. As seen, the efficiency of AμT mechanical wave excitation decreases as intraocular pressure increases.

Because the elastic modulus can potentially change as a function of IOP and propagation direction, so too can the maximum displacement magnitude near the US source since the US intensity, and, hence, the radiation force is kept constant for all measurements. Figure 4 shows that the amplitude of the mechanical displacement wave averaged within the excitation region decreases with IOP at 0° propagation, consistent with previous US-based acoustic radiation force impulse (ARFI) imaging studies in many tissues showing a strong correlation between higher wave speeds and smaller displacements^19^. Combining quantitative maps of the Young’s modulus determined from wave speed measurements, as discussed below, with simultaneous maps of corneal displacement for a known radiation force and corneal thickness can potentially drive biomechanical models to estimate IOP **without** any assumptions about cornea mechanical properties. This approach will be tested in future studies and quantitatively compared to current clinical IOP measurement devices that must assume some average elastic properties for the cornea.

## Discussion

We have shown that reflection-based ARF from air can excite mechanical transverse waves in porcine eye cornea with sufficient displacement amplitude to be tracked with an imaging system even at very low acoustic pressures. We call our method acoustic micro tapping (AμT). The acoustic intensity is many times smaller than safety guidelines used in diagnostic ultrasound [**Methods Section**]. Because both US and OCT are already used extensively in the clinic, there appears to be a straightforward path to translate the entire method (AμT-OCE) into a routine clinical tool.

The proposed method to reconstruct cornea elasticity is of a great importance to current air-puff/tonometry-based methods to estimate IOP. A number of studies have shown that IOP estimates strongly depend on cornea elastic properties^24^,^25^,^26^,^27^,^28^. Here we confirm that cornea elasticity is IOP dependent. Indeed, collagen fibers within soft tissues such as the cornea tend to bear primary mechanical loads. Crimped collagen fibers gradually elongate and interact with the hydrated tissue matrix. This creates a strong non-linearity in the stress-strain relation, i.e., elastic moduli (including elasticity) depend on applied stress^29^,^30^,^31^. Similar effects were observed previously using a contact US approach^32^, and also when an air-puff was used to excite mechanical waves in the cornea^33^.

Here, we have not only demonstrated efficient AμT-based imaging of mechanical waves in biological tissue, but also that the wave propagation speed and displacement amplitude can be measured at each point of the imaged volume. As noted above, if the wave speed can be converted into a quantitative estimate of the elastic (Young’s) modulus, then the combination of modulus, thickness, and displacement maps can be used with an appropriate biomechanical model to image not only the elastic properties but also estimate the IOP **independent** of cornea mechanical properties. This hypothesis will be tested in future controlled studies on both animal and human whole eyes *ex-vivo*.

Because the mode type of generated mechanical waves greatly influences how wave speed measurements are converted into modulus estimates, the appropriate mode must be identified for each application. For the porcine cornea results presented here, the primary mode is a guided (Lamb) wave with significant frequency dispersion over the kHz range given the thickness of the cornea relative to a shear wave wavelength. Consequently, dispersion must be taken into account to produce quantitative measures of the Young’s modulus.

**Figure 5:**
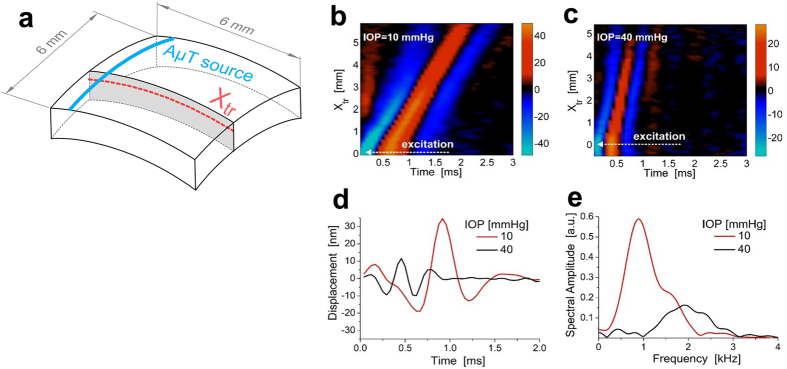
Time and spectral characteristics of the propagating mechanical wave. Two dimensional maps of the temporal profiles (lateral coordinate along the trajectory shown in panel (**a**), *X*_*tr*_, versus time, *t*) at a depth of about 0.1 mm from the surface in porcine eye cornea at different intraocular pressures: (**b**) −IOP = 10 mmHg, (**c**) −IOP = 40 mmHg for propagation at 0°. Typical temporal profiles of transverse mechanical waves (**d**) and their spectra (**e**). Decreased displacement amplitude and a shift of the carrier signal frequency to higher frequencies are observed with increased intraocular pressure.

To understand the role of dispersion in OCE of the cornea, consider a particular propagation trajectory (dashed line in Fig. 5a) and the transient displacement as a function of propagation distance, X_tr_, and time along this path, as shown in Fig. 5b,c at 10 mmHg and 40 mmHg IOP for 0° propagation, respectively. The local slope of the X_tr_-t plots determines the group velocity of the propagating wave. As shown, the group velocity is over two times larger at 40 mmHg IOP than at 10 mmHg IOP.

However, additional information can be extracted. For example, the temporal profile of the displacement at a fixed X_tr_ position (Fig. 5d) is much wider at 10 mmHg IOP than at 40 mmHg. This change is also quite clear in the frequency domain (Fig. 5e), where the center frequency of the signal spectrum shifts significantly (~1 kHz for 10 mmHg versus ~2 kHz for 40 mmHg). Thus, the higher the IOP, the larger the characteristic frequency of the mechanical wave excited for the same AμT source. Like the wave speed and displacement magnitude, the characteristic frequency is related to both the elastic modulus and the IOP.

**Figure 6:**
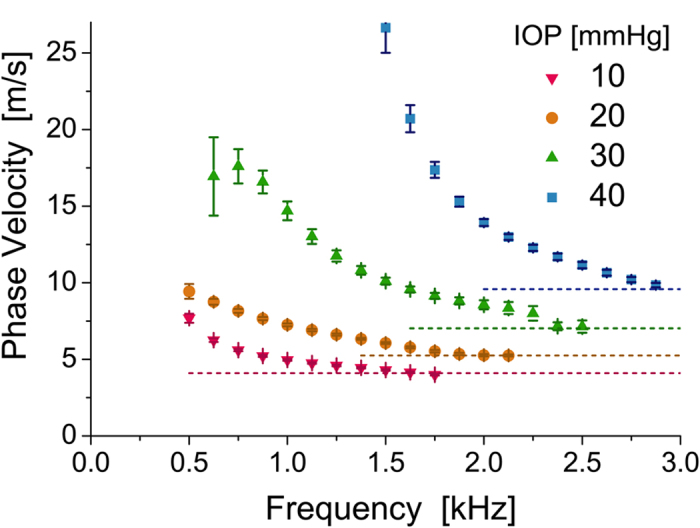
Phase velocity dispersion in *ex-vivo* porcine eye cornea. Temporal profiles of propagating mechanical waves and their subsequent Fourier transform can be used to calculate the velocity of different frequency components, i.e. phase velocity. The phase velocity shows a strong frequency dispersion in the low-frequency range due to the top and bottom boundaries of the cornea, and approaches a high-frequency limit (dashed lines) that differs at different intraocular pressures for propagation at 0°. The high-frequency threshold of phase velocity can be used to evaluate cornea elasticity.

The displacement time waveforms can also be used to estimate the phase velocity, i.e., the phase increment with time, as a function of signal frequency given the broadband character of the propagating waves. Figure 6 shows typical phase velocity frequency dispersion curves obtained for the trajectory illustrated in Fig. 5a and averaged for a region of *X*_*tr*_ between 2 and 3 mm from the AμT source. The same trajectory was used for all IOP over the range of 10–40 mmHg for 0° propagation.

The dispersion is clearly very strong, especially for high IOP. It is determined mostly by boundary conditions and the thickness of the layer^34^,^35^. For the cornea, the mechanical wave in this frequency range is localized primarily within the cornea but leaks inside the eye interior during propagation. Unlike wave propagation in unbounded media where only two propagating modes are present (i.e. longitudinal and shear for an isotropic case), wave propagation in bounded materials supports multiple Lamb modes^34^,^35^, determined by the frequency range and wave excitation conditions. Because only the displacement along the optical beam path is recorded with OCT, mode polarization is also important. A key parameter is the ratio of the layer (cornea) thickness to wavelength of the propagating wave. In our case, this ratio is about 2 and four lower-order modes can exist simultaneously^34^,^35^. Thus, the dispersion curves illustrated in Fig. 6 may contain a few modes, although we selected the most powerful for Fourier analysis.

A detailed analysis of mechanical mode propagation in a bounded medium is complicated, requiring a careful theoretical analysis to account for the eye spherical geometry. This is beyond the scope of this paper, but we will address it in future studies. However, we emphasize a few important points below from the curves illustrated in Fig. 6 that strongly suggest quantitative modulus maps can be obtained with AμT-OCE.

First, any approach using only the group velocity for cornea elasticity assessment, exploited in multiple studies^14^,^36^,^37^,^38^, is not accurate and may lead to erroneous conclusions, especially if low bandwidth signals are considered. Indeed, the group velocity strongly depends on the frequency range or characteristic wavelength. For example, at 1 kHz the group velocity can be twice that at 2 kHz for the primary mode excited here, directly leading to a four-fold difference in estimates of tissue elasticity. Indeed, group velocity based methods using different carrier frequencies and bandwidths will result in different elasticity estimates of the same bounded material.

Full dispersion analysis with broadband waves produced by AμT can overcome these limitations. The main question here is whether it is feasible to use ARF-based imaging (including the noncontact method proposed here) for correctly estimating cornea elasticity. To properly compute the elastic modulus for a bounded medium, the bulk shear (not guided) wave speed is required. Fortunately, the speed of the bulk shear wave is uniquely related to the high frequency limit (dashed lines in Fig. 6), determined by either the speed of the Rayleigh wave (for zero order modes) or the speed of bulk shear wave (for higher order Lamb modes)^34^. Thus, the high frequency limit of the phase velocity must be used instead of the group velocity to produce quantitative estimates of the shear/Young’s modulus in ARF-based elasticity imaging of bounded media such as the cornea. The modulus estimated in this way does not depend on the bandwidth of mechanical waves and is appropriate for biomechanical predictions of near-static deformations in the cornea.

Previous studies suggest that the elastic modulus in the cornea may be anisotropic^39^. Some studies report strong anisotropy^32^,^40^, while others report near isotropic properties^41^. One explanation for this discrepancy could be related with cornea thickness fluctuation^42^. The AμT method can definitely be used to measure anisotropy, but it requires multiple samples for correct statistics. In this study we did not find significant anisotropy (>5%) for all IOP in the range of 10–30 mmHg and all (0°, 45°, 90° and 135°) propagation directions. For 40 mmHg IOP, we found increased wave velocity of ~25% for 135° propagation. Whether this change is related with true anisotropy or a non-linear change of cornea elasticity for such high IOP remains to be determined.

The current AμT-OCE imaging system is a simple proof-of-concept device that can be greatly improved in the near future for clinical applications. For example, the air-coupled ultrasound transducer contains a single element providing a single cylindrical focus to one position. To induce a mechanical wave at a different position, the transducer must be physically moved. This is certainly not optimal.

The current AμT setup can ultimately be replaced with an array of US elements, similar to conventional medical US arrays operating in the low MHz regime. The AμT source can be moved electronically and multiple foci synthesized simultaneously using array processing. Recent work in US shear wave imaging has shown that multiple simultaneous source positions distributed laterally combined with directional filtering of displacement waveforms can greatly increase the size of the tissue volume probed with a single mechanical excitation^43^.

There is also a need to further improve the performance of the PhS-OCT imaging system developed here. The OCE lateral field of view is currently limited to 6 mm × 6 mm, not sufficient to cover the entire cornea. To improve the field of view, both the OCT scanning system and AμT source must be scanned. Note also that 10 signal averages (10 repeated B-scans) are now needed to achieve sufficient signal-to-noise ratio (SNR) for imaging and characterization of propagating mechanical waves in the cornea, which increases the time needed for data acquisition.

In addition, the OCT system can leverage recent advances in laser technology to greatly increase the 3-D scan rate. The latest generation of swept source lasers providing A-Scan rates over 20 MHz, with multi-beam configuration, can potentially increase scan rates by a factor of ten^44^. If the noise sources in the present system can be overcome and noise reduction techniques applied to the higher sweep-rate lasers, then the full 3-D volume of the cornea may be addressed in less than 1 sec. Matching the air-coupled array approach with faster OCT scan rates could enable real-time, or near real-time, OCE of the cornea. This is an exciting prospect for ophthalmology, and other potential applications in biomedicine.

Quantitative elastic modulus maps offer exciting opportunities to better understand and evaluate corneal biomechanics in diseases (such as keratoconus) and in surgical planning (refractive surgery and corneal transplant surgeries). Furthermore, AμT-OCE can provide new insights into the role of elasticity in many ophthalmic conditions such as ocular surface tumor characterization, scleral elasticity and myopia, and risk factors in glaucoma progression. Also, we will use this new technology on eye bank cornea to study changes in corneal elasticity induced by interventions such as Lasik surgeries.

The method is not limited to the cornea, however. It can be easily adopted for many medical applications where optical methods are currently used, such as characterization of skin elasticity or mapping the elastic properties of tissue biopsies. The method can easily meet all safety requirements, and is very convenient for clinical use because it is absolutely non-contact and potentially real time (**Methods Section**). To our knowledge, this is the first experimental demonstration of non-contact ARF-based generation of broad bandwidth mechanical waves in soft tissues; and this is the first experimental demonstration of a fully non-contact and non-invasive method for soft media elasticity characterization combining air-coupled ultrasound and PhS-OCT.

Finally, medical applications are the primary focus for AμT, but many non-medical uses are also possible. For example, the elasticity of any soft substance, especially fragile materials, can be characterized because no contact is made with the sample. In this context, we define a soft material as nearly incompressible (i.e., Poisson’s ratio approaching 0.5), where the bulk modulus determining the sound speed is several orders of magnitude larger than the shear modulus determining the speed of transverse waves in the medium. In most cases, the acoustic impedance of a soft medium is much different than that of air, so AμT can efficiently launch transverse mechanical ways in the medium without contact. A number of imaging approaches can potentially monitor mechanical wave propagation to probe the elastic properties of that medium. In this way, AμT can become a routine tool to probe the elastic properties of soft materials, especially delicate samples easily damaged by contact or material systems where contact may change the elastic properties of the sample.

## Methods

### Soft tissue under study

Porcine eye was enucleated immediately after death, and OCE measurements were performed within 20 hours after enucleating. Before performing any measurements, it was kept in a refrigerator at 4 °C within a chamber, surrounded by cotton soaked in physiological saline. During measurement, the whole porcine eyeball was placed into a custom-built holder with a half-sphere cup and moisturized cotton to provide an *in situ* environment. The eye globe was oriented with cornea side up and the optic axis vertical. The OCE scanning beam paralleled the optic axis.

A 23 G needle was inserted through the sclera and connected to an infusion reservoir at the other end. The IOP was controlled by adjusting the height of an infusion reservoir.

All tissue samples used in these studies were acquired through an abattoir. All methods were carried out in accordance with institutional guidelines and regulations for studies on tissue. All experimental protocols followed standard operating procedure established by the University of Washington for the use of animal tissue acquired from an abattoir in research studies.

### Acoustic micro-tapping to excite mechanical waves in soft tissue

Acoustic radiation force (ARF) is an efficient method to excite shear acoustic waves in soft tissue. When a pump US beam is focused within a medium, enhanced acoustic absorption in the focal area results in partial conversion of the beam energy into a transient tissue displacement parallel to the beam direction but propagating laterally, creating shear acoustic waves^2^,^3^,^4^,^19^,^21^. Propagating bulk shear waves can be tracked with an imaging system to locally estimate the wave speed and map the shear (Young’s) elastic modulus. This approach is now widely used^17^,^18^,^45^,^46^,^47^ because the shear wave source can be created remotely near the region of interest in a clinical scan. Unfortunately, however, it requires direct contact with the tissue under study and is not appropriate for applications where physical contact is not desired (e.g., ophthalmology).

At the interface between two media, ARF works in a different manner to launch shear waves. Due to conservation of the mechanical impulse (i.e., Newton’s third law for a continuous mechanical medium), a partial reflection of the pump US beam from the interface will induce a “push” towards this interface with radiation pressure *P* (force per unit area) equivalent to^20^,^21^,^48^


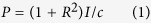


where *R* is the reflection coefficient at the interface, *I* is the acoustic intensity [Watts/m^2^] and *c* is the sound speed of the first medium. When acoustic impedances (products of density and US speed) of the connected media are close to each other, reflection-based ARF is negligible.

The situation dramatically changes when one of the media is air. If the pump beam is directed from tissue to the medium/air interface, the tissue volume is partially released into air. For liquids, this effect can create an acoustic fountain^49^,^50^,^51^. For the opposite case where the pump acoustic beam is directed from the air to the air/medium interface, the reflection coefficient is nearly one (*R* ≅ 1) and, therefore, nearly all acoustic intensity is converted into radiation pressure.

When the tissue relaxation time from the induced ARF is longer than the tapping time, the characteristic wavelength of the generated mechanical wave is determined by the width of the AμT source in the direction of mechanical wave propagation. In other words, the characteristic wavelength of the generated mechanical wave is determined by the tapping source spot at the tissue surface. The carrier frequency of the tapping source, around 1 MHz in this study, does not influence the wavelength of the generated mechanical wave at all because ARF-based conversion is defined by the pump beam intensity envelop. The carrier frequency of the pump beam determines the ultimate focal spot and, hence, the size of the mechanical wave source.

Our approach uses a specially designed air-coupled piezoelectric transducer to launch through air an US beam focused on the air-medium interface. We designed and built a cylindrically focused air-coupled piezoceramic transducer that can efficiently transfer a 1 MHz US pulse through air to the tissue surface. The transducer has a matching layer (a 0.45 μm pore size nylon membrane filter, Cat. No. 7404–004, “GE Healthcare UK Limited”, UK) bonded to the transducer surface with a silicone adhesive. The piezoceramic element was cut from a piezo-cylinder (cat.# 42–1041, APC International Ltd, USA) to create a 9 mm long section of 75 degrees. The outer and inner diameters of the transducer are 30 mm and 26 mm, respectively.

Supplementary Figure S1 presents an US intensity field emitted by the transducer into air, as measured with a 0.4 mm needle hydrophone (Part # HNC-0400, Onda, USA) directly in air. The transducer is cylindrical focused and therefore its maximum intensity area is localized into a strip in the (XY) plane (see Supplementary Fig. S1b). The length of this strip determines the length of the AμT source. The distribution of the transducer field in the (XZ) plane (see Supplementary Fig. S1c) determines its actual focal zone; the width of the focal zone in the X direction defines the AμT source width shown in Supplementary Fig. S1d. When the tissue relaxation time from induced tapping is shorter than the tapping time, the characteristic wavelength of the generated mechanical wave is determined by the width of the AμT source in the direction of mechanical wave propagation. Note that this wavelength defines the ultimate in-plane imaging resolution.

The transducer was tilted to the porcine eye surface by about 45 degrees to its normal to not block the OCT beam tracking the propagating mechanical wave. The cornea surface was in the transducer focus. It was pushed using an US burst with repetition period of 3 ms to synchronize the resultant mechanical wave with the B-scan rate of the PhS-OCT imaging system. The burst was a linear chirp 250 V peak to peak in amplitude and 200 μs in duration, where a chirp was used to minimize potential standing wave effects between the transducer and the phantom surface. The bandwidth of the driving signal ranged from 0.95 MHz to 1.05 MHz (i.e., chirp has a time-bandwidth product of approximately 20). The pressure amplitude in the transducer focus (peak acoustic pressure) was measured to be about 7 kPa.

At this peak pressure level, the spatial peak pulse average intensity, I_SPPA_, is about 1 W/cm^2^ in air. Because a major part of the acoustic intensity is reflected at the boundary (99.9%), the I_SPPA_ within the cornea is estimated to be less than 1 mW/cm^2^, representing only 10^−4^ of the 28 W/cm^2^ exposure guideline for ophthalmic imaging^52^. Even at 100% duty cycle for ultrasound exposure (in this experiment a duty cycle of about 7% was used), the spatial peak time average intensity, I_SPTA_, is still two orders of magnitude below exposure limits. Clearly, AμT should not pose any safety issues for clinical applications.

### Ultra-fast frame rate 4D PhS-OCT imaging system

Based on its high-resolution and high-sensitivity to motion, phase-sensitive OCT (PhS-OCT) is a very efficient imaging modality for dynamic microscale elastography (or OCE)^53^. A conventional M-B scan has been widely adopted for OCE imaging^9^,^10^,^12^,^36^. In this mode, multiple A-scans are recorded for a fixed spatial position at different time instants to form one M-scan at a frame rate sufficient to capture the dynamics of shear wave propagation. For the range of mechanical waves considered here, at least a 10 kHz frame rate is usually required. M-scans are taken across the 2-D image plane, while the excitation is repeated for each M-scan. However, recording multiple M-B scans is necessary for volumetric (or 4D, i.e. 3D plus time) OCE imaging, which is time consuming for *in-vivo* applications.

Recent developments in high-speed swept laser sources and phase-stabilization techniques have enabled MHz level A-line rate imaging with outstanding phase stability for PhS-OCT^15^. Thus, the entire B-scan (instead of an A-scan) can be recorded for just one mechanical wave excitation and overall data acquisition time can be reduced dramatically.

To track the tissue displacement in 4D with high sensitivity and high resolution, we developed a fast PhS-OCT system (Supplementary Fig. S2). A commercially available high-speed FDML swept laser (Optores GmbH, Germany) was employed as the OCT light source capable of 1.62 MHz sweep repetition rate over a spectral bandwidth of 110 nm centered at 1308 nm. The output light was coupled into the PhS-OCT system via a 90/10 fiber coupler with 10% of light routed to the reference arm. The rest went to the sample arm, where it was further split into a calibration arm and the sample arm via another 99/1 fiber coupler. The calibration arm combined with the reference arm formed a slave interferometer providing a reference signal to quickly calibrate spectral interferograms for OCT signal reconstruction. Back-scattered light from the sample arm and light from the reference arm each pass through an optical circulator and are then combined by a 50/50 fiber coupler, forming a Mach-Zehnder interferometer that generates the OCT signal. The signal is detected by a high-speed balanced photo detector (PDB480C-AC, Thorlabs Inc., USA), and subsequently digitized by an analog to digital converter card (ATS9373, AlazarTech, Canada) at 3.6 GS/s. Captured data are transferred to a host PC through PCIe bus, and finally processed for real-time preview, or stored for later processing.

The OCT probe (see Fig. 1) in the sample arm contains a dual-axis galvanometer scanner and an object lens with a 35 mm focal length. The fast-axis scanner was resonant (Electro-Optical Products Corp., USA). It was driven by a triangle waveform (7950 Hz) and synchronized by a phase locked loop (PLL) module on the FDML laser.

We used both directions of the scanned, focused sample beam to produce B-Scans at a rate of 15,900 frames per second. For each B-scan, there were 102 A-scans. These parameters parallel those described in previous studies of phase-stabilization strategies^15^. The slow axis was driven by a galvo motor (6215 H, Cambridge Technology, USA) to sweep a full volume of B-Scans. The axial (in-depth) resolution was measured to be ~15 μm in air; the lateral resolution is 58.8 μm over the entire scan area (lateral field of view) of 6 mm × 6 mm. The system ranging distance was up to 4 mm.

The scan protocol and synchronization of AμT was controlled by an analog output device (PCI6713, National Instruments, USA). For each AμT excitation, the 4-D scanning protocol repeated 48 B-scans separated by 62.5 μs to collect a time course of B-scans, i.e., full M-B scan taking just 3 ms. There were 102 AμT excitations, each synchronized with the first B-scan on each image plane, resulting in 102 image planes to cover the entire 3-D region of interest in only 0.3 sec. The procedure was repeated 10 times to improve the signal-to-noise ratio, resulting in a total data acquisition time of 3 sec.

The displacement was computed from the phase of the OCT signal, as described previously^53^. For each volume, the displacement field was extracted to visualize wave propagation, as shown in Supplementary Movie S3. Distortion from non-linear resonant scanning was corrected by spatial re-sampling, and sample time differences between beams within one B-scan were corrected by temporal re-sampling. Potential surface ripple artifacts were also suppressed using an automatic surface detection method described in a previous study^54^.

## Additional Information

**How to cite this article**: Ambroziński, *et al*. Acoustic micro-tapping for non-contact 4D imaging of tissue elasticity. *Sci. Rep.* **6**, 38967; doi: 10.1038/srep38967 (2016).

**Publisher's note:** Springer Nature remains neutral with regard to jurisdictional claims in published maps and institutional affiliations.

## Supplementary Material

Supplementary Figure File

Supplementary Movie S3
