# Supplementary material for: Acoustic micro-tapping for non-contact 4D imaging of tissue elasticity
Source: Sci Rep. 2016 Dec 23;6:38967. doi: 10.1038/srep38967 (PMC5180181; doi:10.1038/srep38967)
Supplement: Supplementary Figure File [file srep38967-s1.pdf]

Supplementary Information – Supplementary Figures

Acoustic micro-tapping for non-contact 4D imaging of tissue elasticity

+Łukasz Ambroziński[1,2], +Shaozhen Song[1], +Soon Joon Yoon[1], *Ivan Pelivanov[1,3], David Li[1,4], Liang Gao[1], Tueng T. Shen[1,5], Ruikang K. Wang[1,5] & *Matthew O'Donnell[1]

[1] Department of Bioengineering, University of Washington, Seattle, WA 98195, USA

[2] AGH University of Science and Technology, Krakow, Poland

[3] Faculty of Physics, Moscow State University, Moscow, 119991, Russia

[4] Department of Chemical Engineering, University of Washington, Seattle, WA 98195, USA

[5] Department of Ophthalmology, University of Washington, Seattle, WA 98104, USA

* Corresponding authors are Dr. Ivan Pelivanov, e-mail: ivanp3@uw.edu; and Dr. Matthew O'Donnell, e-mail: odonnel@uw.edu.

+ First three authors (Łukasz Ambroziński, Shaozhen Song, Soon Joon Yoon) made equal contributions to the paper.

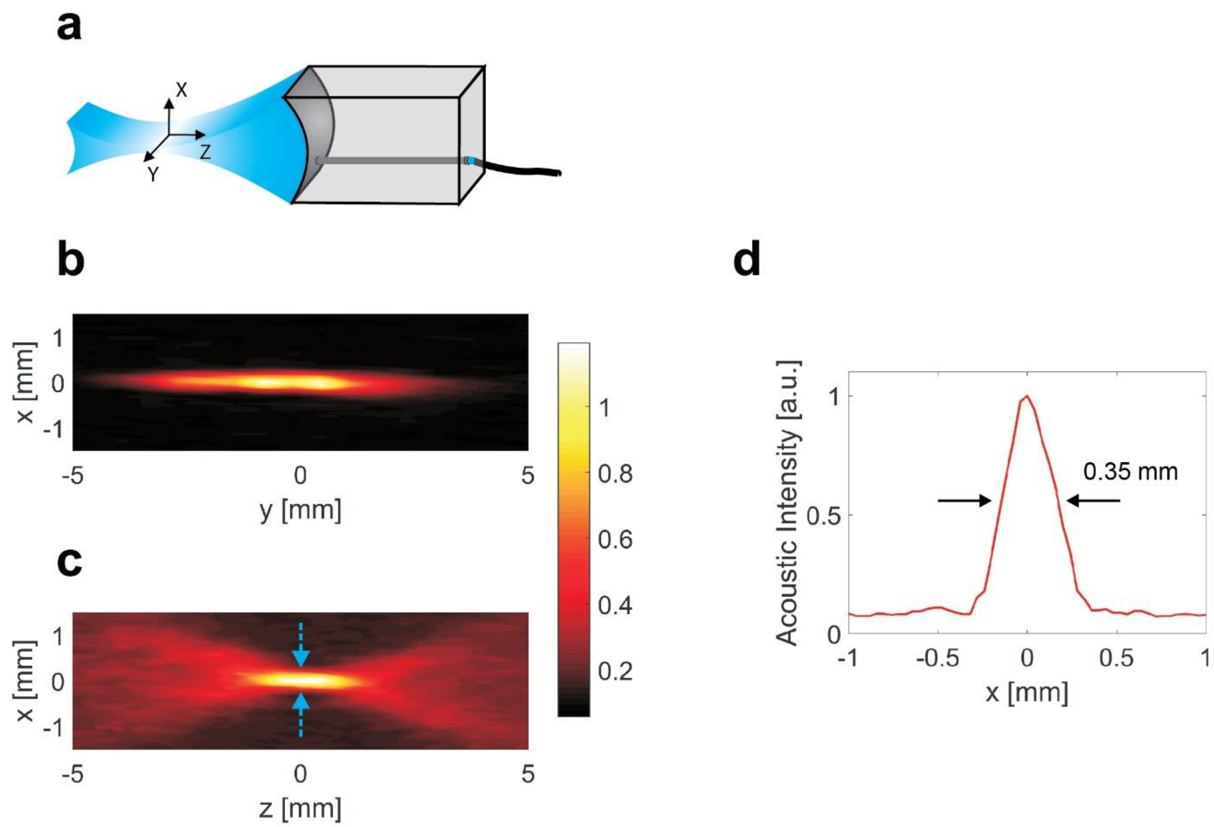

Supplementary Figure S1. (a) - Arrangement of air-coupled transducer in the coordinate system defined with respect to the transducer focal point. Air-coupled transducer focal zone shown in terms of acoustic intensity (pressure squared) on coordinate: (b) – (XY) plane, (c) – (XZ) plane. (d) - Profile of the acoustic intensity across X-axis.

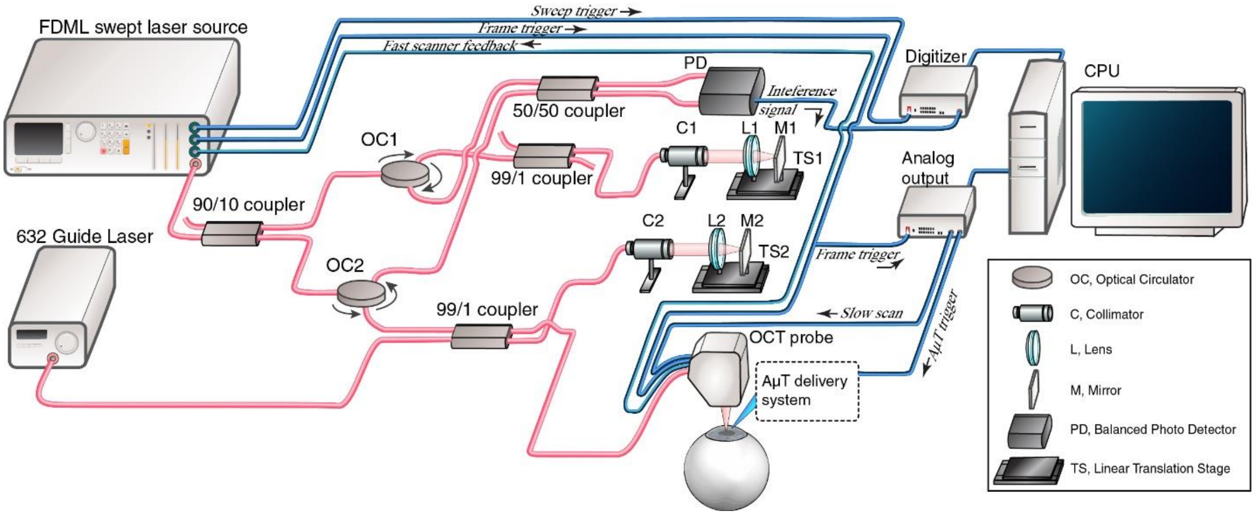

Supplementary Figure S2. Schematic diagram of the high-speed OCT imaging system setup, where an ultra-high-speed FDML swept laser is utilized as the OCT light source. The source was tuned to a center wavelength of 1308 nm with a spectral bandwidth of 110 nm at ~1.62 MHz sweep rate. Ninety percent (90%) of the energy is delivered to the sample arm, and 10% is directed to the reference arm (C1, L1, M1). Using another fiber coupler, 1% of sample arm light is delivered to a re-calibration arm (C2, L2, M2), forming a slave interferometer with the reference light for quick re-calibration of spectral interference signals. The slave interferometer is disabled during imaging. A visible red laser (632 nm) is used as a guide beam to indicate the OCT scan beam position at the sample. The light returned from sample and reference arms are combined (interfered) at a 50/50 coupler via two optical circulators (OC1 and OC2), then the interference signal is converted to an electronic signal by a high-speed, balanced photo detector. A high-speed digitizer samples the converted interference signal at 3.6 Gs/S. An analog output device is utilized as the waveform generator to control galvanometer scanning, as well as triggering the AµT delivery system. The frame trigger signal generated by the FDML laser source is used to synchronize the OCT scan, and the position feedback signal is sent back to the FDML laser source to stabilize the fast scanner's phase. More details for the OCT probe are shown in **Fig. 1**. The fast-axis scanner is resonant, operating at 7950

Hz. The effective B-frame rate in the experiment was 15,900 fps, where each B-scan contains 102 A-lines. Forty-eight (48) images are taken on each image plane, and 102 image planes are acquired to form a 4D scan. Thus, each 4D scan takes approximately 0.3 second. Ten repeated measurements are acquired and averaged to achieve sufficient SNR for displacement imaging for this experimental setup. The equivalent spatial resolution in transverse directions is 58.8 μm, and the lateral scan range is 6×6 mm (i.e., the field of view), with a range capability of ~ 4 mm.

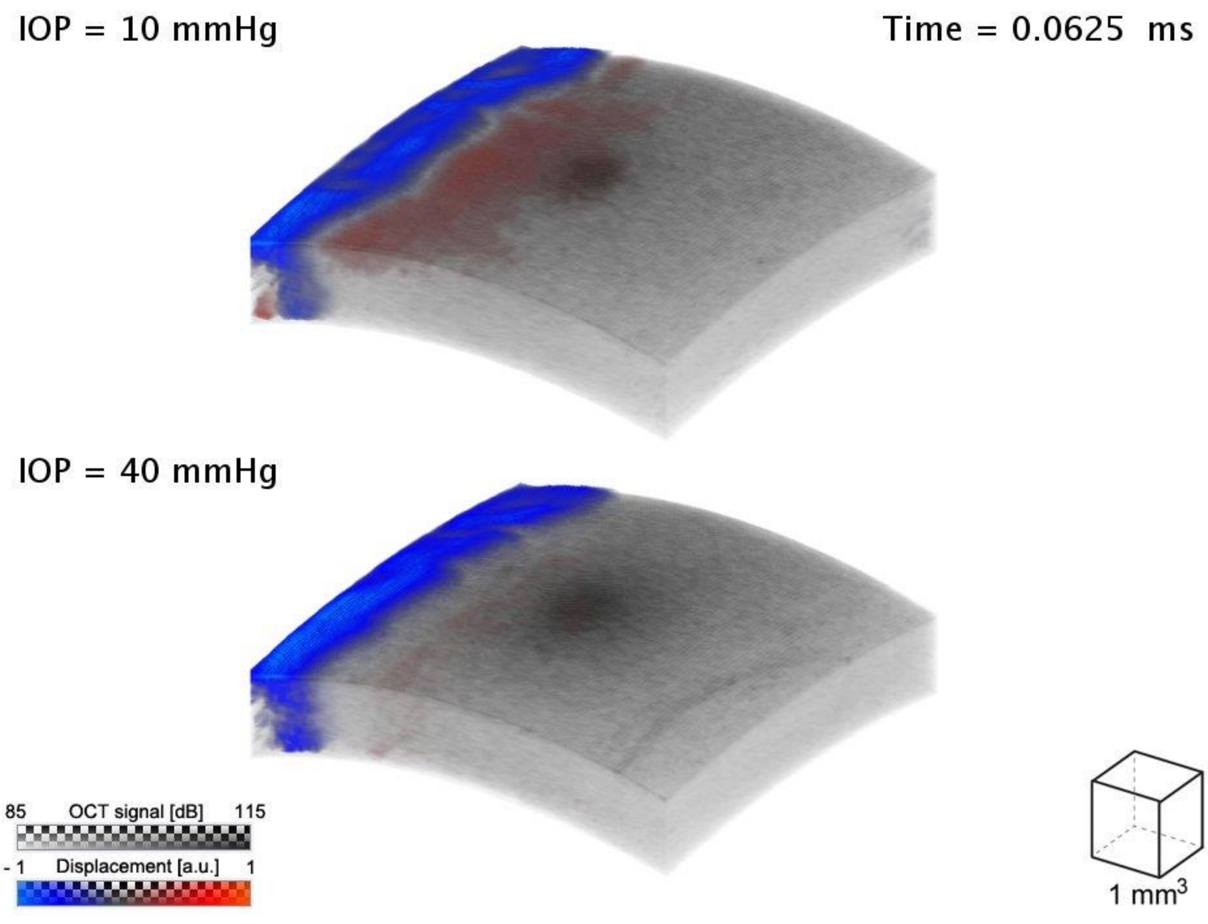

Supplementary Movie S3. (first snapshot is shown here only; see **Supplementary_Movie_S3.mp4** for the entire movie)

4D mechanical wave propagation in ex-vivo porcine eye cornea at different ocular pressures: 10 mmHg (upper panel) and 40 mmHg (lower panel) for propagation at 0°. The displacement amplitudes are normalized for both data sets by their maxima in the excitation area (62 nm and 34 nm, respectively). 4D data are acquired at 15,900 volumes per second. Video played at 16 frames per second. The bottom of the colorbar indicates the voxel color, and the top indicates the transparency applied to any voxel, on a checkerboard background.
